# Biliary reconstruction with localized creation: One case report of repairing bile duct injury and defect with autografts

**DOI:** 10.1016/j.ijscr.2024.109597

**Published:** 2024-04-02

**Authors:** Feibo Zheng, Yuqing Zhang, Liang Ha, Jipeng Xia, Yunfeng Cui

**Affiliations:** a Department of Hepatobiliary and Pancreatic Surgery, Department of Surgery, Tianjin Nankai Hospital, Nankai Clinical School of Medicine, Tianjin Medical University, Tianjin 300100, China; b Department of Surgery, Tianjin Occupational Diseases Precaution and Therapeutic Hospital, Tianjin 300011, China

**Keywords:** Bile duct injury, Mirizzi syndrome, Autograft, Hepatobiliary, Case report

## Abstract

**Introduction:**

Bile duct injuries caused by any reason are a disaster for patients and pose a significant psychological and technical challenge for surgeons. The use of Ligamentum teres hepatis and gallbladder flap as autografts is showing promising results in the repair of bile duct injury.

**Case presentation:**

This article presents a challenging case of a patient with Mirizzi syndrome who experienced a complex bile duct defect and injury during cholecystectomy. We describe the successful reconstruction of the bile duct using ligamentum teres hepatis and remnant gallbladder flap simultaneously.

**Discussion:**

Ligamentum teres hepatis and remnant gallbladder flap are ideal repair materials for repairing and reconstructing bile duct injuries due to their easy availability, good tissue compatibility, and low incidence of postoperative complications. It is essential to seek the assistance of an experienced biliary surgeon when bile duct injury occurs during operation.

**Conclusion:**

Ligamentum teres hepatis and gallbladder flap, as suitable autologous tissues, are viable options for repairing bile duct injuries and defects.

## Highlights

• Ligamentum teres hepatis and remnant gallbladder flap are viable autologous tissues for repairing bile duct injuries and defects.
• The innovative use of these autografts in a challenging case of Mirizzi syndrome highlights the potential of individualized surgical approaches and the importance of experienced biliary surgeons.
• The gold standard treatment for major bile duct injuries may lead to long-term complications, emphasizing the need for alternative approaches such as autograft reconstruction.

## 1 Introduction

Bile duct injury (BDI) is a serious complication that can occur during cholecystectomy or other hepatobiliary surgeries, potentially leading to significant morbidity and mortality if not managed appropriately. Various surgical techniques have been developed to address such injuries, including the use of different tissue flaps for reconstruction. Ligamentum teres hepatis (LTH), also known as the round ligament of the liver, as well as the gallbladder flap, have been proposed as suitable autologous tissues for bile duct reconstruction due to their proximity to the biliary system and their potential for regeneration. Previous studies have shown promising results, indicating their viability for repairing bile duct injuries and defects [[1], [2], [3]].

In this case report, we present a challenging case of a patient with Mirizzi syndrome (MS) who experienced a complex bile duct defect and injury during cholecystectomy. We describe the successful reconstruction of the bile duct using LTH and the remnant gallbladder flap simultaneously for the first time. This case highlights the importance of innovative surgical techniques and the potential of autologous grafts in the management of bile duct injuries. The aim of this report is to contribute to the existing literature on bile duct reconstruction and provide insights into the use of LTH and the residual gallbladder flap as a feasible option for addressing complex bile duct injuries and defects. This case underscores the significance of individualized surgical approaches in the management of hepatobiliary complications and the potential for favorable outcomes with the utilization of autologous tissue for reconstruction. This case report has been reported in line with the SCARE Criteria [4].

## 2 Case presentation

**Fig. 1:**
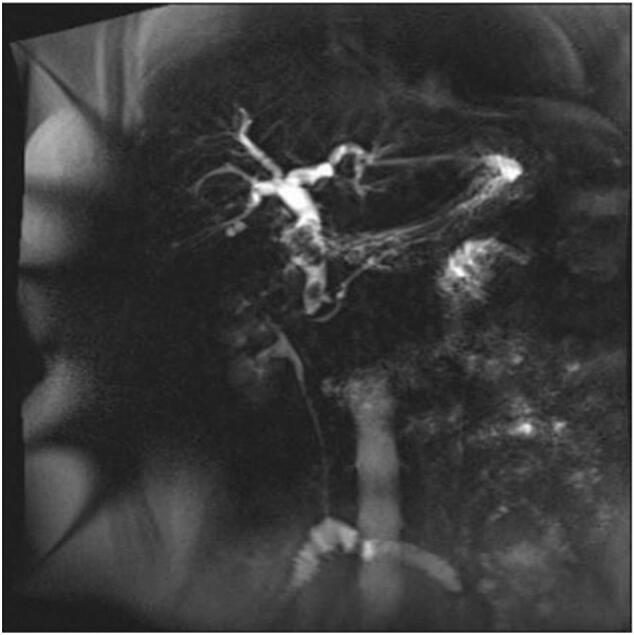
Preoperative MRCP: dilatation of intrahepatic and extrahepatic bile ducts, with gallbladder neck impaction by gallstones and CBD stone.

**Fig. 2:**
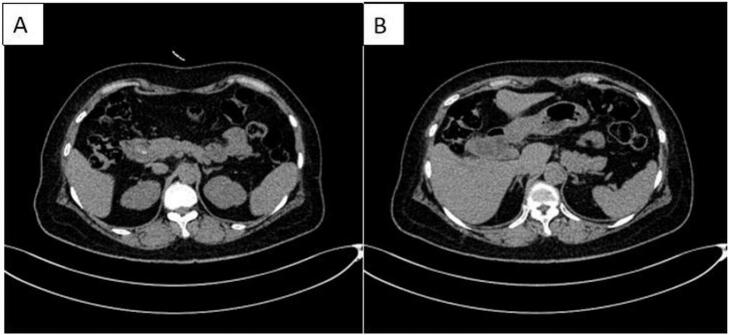
Preoperative CT A:The common bile duct is obstructed by a stone and dilated, with slight thickening of the lower end wall; B:bile stasis and cholecystitis, with the possibility of gallbladder neck stones.

A 64-year-old woman presented with epigastric pain and distension for 10 days more was referred to our hospital. A recent Magnetic Resonance Cholangiopancreatography (MRCP) from another hospital revealed multiple stones in the gallbladder and common bile duct (CBD) along with cholecystitis (Fig. 1). The patient had a history of high blood pressure. On arrival, vital signs were unremarkable except for a mild elevation in blood pressure to 144/91 mmHg. Physical examination revealed a positive Murphy sign and abdominal swelling. Laboratory tests showed elevated gamma glutamyl transferase at 270 U/L (range 11-50 U/L), alanine aminotransferase at 81 U/L (range 5-40 U/L), and alkaline phosphatase at 149 U/L (range 34-114 U/L). Total bilirubin, direct bilirubin, and indirect bilirubin levels were normal. A computed tomography (CT) scan of the abdomen indicated CBD stones, slight thickening of the lower wall of the CBD, dilation of both intrahepatic and extrahepatic bile ducts, bile stasis, and cholecystitis, with a possibility of gallbladder neck stones (Fig. 2). Combining the findings from the MRCP and CT, the patient was diagnosed with common bile duct stones with cholangitis, and gallbladder stones with cholecystitis. Following adequate preoperative preparation, laparoscopic cholecystectomy and laparoscopic common bile duct exploration were planned to be performed under general anesthesia on the third day of admission.

**Fig. 3:**
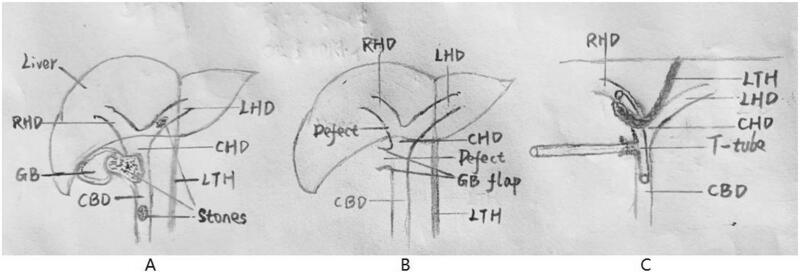
Operative hand-drawing A:Preoperative scenario a huge stone was impacted at the junction of GB and CBD; a stone at the lower end of the CBD, a stone at the LHD. B:After subtotal cholecystectomy and exploration of the bile duct with choledochoscopy stones were extracted and two defect sites left. C:Bile duct reconstruction:RHD defect was repaired with LTH, CBD defect was repaired with remnant GB flap, T-tube was inserted and the upper end of the short arm passing through the defect of RHD. GB:gallbladder CBD:common bile duct CHD:common hepatic duct LHD:left hepatic duct RHD:right hepatic duct LTH:ligamentum teres hepatis.

During the surgery, extensive adhesions were found between the gallbladder and surrounding tissues. The gallbladder appeared atrophic with thickened, hardened walls, measuring approximately 5 cm*2 cm. A stone was impacted at the neck of the gallbladder, with the upper part of the stone invading the serosal layer of the gallbladder wall and the lower part closely fused with the common bile duct (Fig. 3A). Due to unclear Calot's triangle and poor local anatomical structure, it was decided to proceed with open surgery. After laparotomy, a stone with a diameter of approximately 2 cm was extracted, and a cholecystocholedochal fistula was identified. According to the classification of Mirizzi syndrome by Csendes, this case was classified as type III [5], and the defect in the common bile duct was approximately 1.8 cm. Exploration of the bile duct was performed through the defect, and one stone each was found at the lower end of the common bile duct and the left hepatic duct (Fig. 3A). All stones were retrieved using a basket. Subtotal cholecystectomy and the fundus-first (top-down) approach [6] were initially considered and then used. Unfortunately, the lateral section of the right hepatic duct and the confluent section of the common hepatic duct were damaged, resulting in a 1.5 cm defect with bile outflow (Fig. 3B).

Faced with the scarred duct, an experienced hepatobiliary surgeon was called in for an emergency procedure. Initially, he utilized the remaining gallbladder flap along with the preserved cystic artery to mend the common bile duct defect: detaching from the starting point of the cystic artery to create a gallbladder flap with the cystic artery serving as the pedicle, matching the size and shape of the defect, and then using the starting point of the cystic artery as a rotational arc to flip the gallbladder flap over the defect, while also leaving an appropriate gap for the insertion of a T-tube. Additionally, he used LTH to repair the defect on the lateral right hepatic duct: cutting and suturing the LTH section near the umbilical side to the end of the abdominal wall, trimming the pedicle of LTH, and intermittently suturing its serosal surface to the damaged bile duct sidewalls with 6–0 absorbable suture. A T-tube support drain was placed inside the defect of the common bile duct, with the upper end of the short arm passing through the defect of the right hepatic duct, and the T-tube was secured with 4–0 barbed suture (Fig. 3C).

**Fig. 4:**
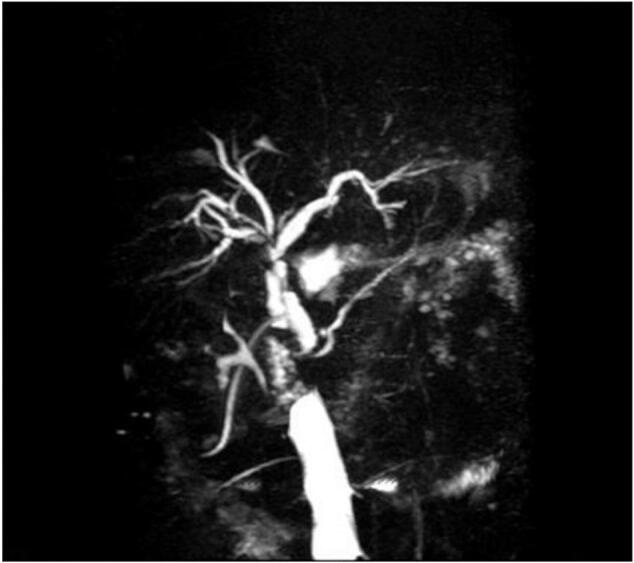
MRCP at 6 months postoperatively: no dilation of the intrahepatic and extrahepatic bile ducts, the diameter of the common bile duct was approximately 7 mm,a tubular shadow were observed in the area from the right hepatic duct to the extrahepatic bile duct.

Following the examination that revealed no significant bleeding in the gallbladder bed, one abdominal drainage tube was placed in the gallbladder bed, and another was placed in the foramen of Winslow. Postoperatively, the gallbladder specimen was sent for routine pathological examination, which resulted in a diagnosis of a subacute attack of chronic cholecystitis with extensive mucosal damage and partial outer membrane, along with a small amount of liver tissue. The patient was discharged on the 15th day postoperatively with a T-tube. Six months later, a follow-up MRCP (Fig. 4) indicated no dilation of the intrahepatic and extrahepatic bile ducts, with the diameter of the common bile duct measuring approximately 7 mm. Additionally, a tubular shadow and filling defects (referred to as a “flow void” effect on MRCP [7]) were observed in the area from the right hepatic duct to the extrahepatic bile duct. Laboratory tests showed normal liver enzymes and bilirubin levels. Consequently, the T-tube was removed. To date, the postoperative outcomes for the patient were favorable, with no serious events observed.

## 3 Discussion

BDI in one of the most common complications of hepatobiliary surgery,often occurring in cholecystectomy, especially in difficult cholecystectomy due to Mirizzi syndrome. For major BDIs, an urgent surgical repair with bilioenteric anastomosis Roux-en-Y hepaticojejunostomy represents the gold standard treatment [8]. Its biggest advantage is that it can fully free the biliary intestinal loop to reduce the tension of the bilioenteric anastomosis site, while utilizing the anterograde peristalsis of the loop to prevent reflux of intestinal contents. However, this procedure alters the normal anatomical structure of both the biliary tract and gastrointestinal tract, leading to a high incidence of long-term reflux cholangitis, anastomotic stenosis and other complications that seriously affect patients' postoperative quality of life. As for the technique of end-to-end anastomosis of bile duct, if the defect is not large and the ends are in good condition, it is feasible to have primary anastomosis. However, due to the severe BDI with large defect and local inflammation and edema in this case, this approach was not being used.

Instead, we successfully used both a pedicle gallbladder flap and round ligament for reconstructing bile duct injuries. The pedicled gallbladder flap originates from the same embryonic origin as bile duct tissue and contains mucosa which can be used for tissue repair while ensuring long-term survival without degeneration that could lead to recurrent bile duct strictures. Additionally, since it belongs to extrahepatic bile duct tissue, it can perform bile transport function well by adapting to physicochemical stimulation without causing significant inflammatory reactions.

The ligamentum teres hepatis is a ligament that forms part of the free edge of the falciform ligament of the liver, connecting it to the umbilicus. It is the remnant of the left umbilical vein during embryonic development, which degenerates to fibrous tissue after birth. Its upper edge connects with the falciform ligament near the bile duct, making it easy to harvest and separate. With its rich blood supply from branches of the right hepatic artery and left portal vein, the LTH is an ideal autologous graft for repairing bile duct defects. During the repair procedure, it's crucial to preserve an adequate length of the ligament based on the location and area of the bile duct defect, ensuring sufficient blood supply and minimal tension at the anastomosis site. Additionally, maintaining T-tube support for at least 6 months is essential for successful healing. Currently, there are few cases of autografts repair of BDIs. More research reports are needed to further clarify its effectiveness and safety.

It's worth emphasizing that the repair procedure should be performed by experienced biliary surgeons. Surgeons specializing in the repair of bile duct injuries have better results than those with less experience [9].

## 4 Conclusion

Using the LTH and gallbladder flap as viable autologous tissues offers promising options for repairing bile duct injuries and defects. Experienced biliary surgeons play a crucial role in minimizing the risk of BDI and ensuring a higher success rate of bile duct reconstruction.

## Consent

Full written consent was obtained from the patient for publication and any accompanying images. A copy of the written consent is available for review by the Editor-in-Chief of this journal on request.

## Ethical approval

This paper was exempt from ethics from out institution as this was a case report and full written consent was obtained from the patient.

## Funding

This report was supported by the 10.13039/501100006606Tianjin Natural Science Foundation key project (21JCZDJC00550), and Tianjin 131 innovative talent team, innovation team for diagnosis and treatment of acute abdomen related to biliary and pancreatic diseases (201938).

## Author contribution

Dr Feibo Zheng – writing the manuscript, editing the manuscript, collection of imaging, patient care and follow-up.

Dr. Yuqing Zhan g's contribution is equal to that of Dr. Feibo Zheng.

Dr Liang Ha and Jipeng Xia – patient care, case study imaging procurement, reviewed and edited the manuscript.

Dr Yunfeng Cui – the experienced surgeon in this article, provided and supervise the case, reviewed and edited the manuscript.

All authors read and approved the final manuscript.

## Guarantor

Yunfeng Cui

## Research registration number

1.Name of the registry:NA

2.Unique identifying number or registration ID:NA

3.Hyperlink to your specific registration (must be publicly accessible and will be checked): NA

## Conflict of interest statement

There are no conflicts of interest for the authors.
